# Office Visitors

**Published:** 1906-09

**Authors:** 


### OFFICE VISITORS.

It was years ago before the advent of cocaine for local anaesthesia. We were summering at a small hotel in the mountain region of the Old North State and had fixed up in a corner of our bedroom, before the window a split-bottomed arm-chair and a few operating instruments on a box on top of a low table and had let it be known at the postoffice that we were prepared for dental service to the country at large. We had climbed the neighboring peaks and visited all the nearby falls and had settled down to the routine of the summer boarder—sleeping, eating, reading, lounging around and about, swinging in the hamocks and we awaited business. It came slowly and we had concluded that with the exception of a few city people, boarding in the hotel, that patrons would be scarce in this region of hillsides and cones and so it proved. The dwellers of the slanting land "didn't want nothin' did but tooth--pullin'" A couple came in one day, man and wife. She wanted the "toothdentis' ter pull out all them old rotten snags in my mouth and put me in er new set, and I want yer ter pull em 'dout killin' me." The only approach to an anaesthetic we had was a bottle of "blockade whiskey," otherwise "mountain dew," that we had run across in our perambulations and "found in a stump." so we allowed her to imbibe very freely. When we found that she was "feelin' the licer pretty smart" we commenced operations. Stimulated with the alcoholic and previously nervous as to the expected pain—magnified by imagination into something terrible, she gave vent to most excruciating yells and heart breaking moans. We were on the point of telling her if she did not control herself, we would be obliged to dismiss her, when we heard a lumbering sound back of us. In a moment she was out of the chair with bloody hands and mouth, exclaiming, "Lord God if there hain't mf ole man fell inter er faint.

Ketch er holt er him, Doctor ,an git im on ther bed. Gimme that er basin er water, whars yer licker, git er swaller down im.'' It was all done and over in a few minutes. She flew around like an expert trained dental nurse bringing a patient to—forgetting for the while all her tumultuous suffering (?). When the husband had recovered and sat up she explained. ''Yes he's poorly, ben sick, but he's faintified anyhow. Kaint stan' ther site er beloved an I recken my takin on, kinder sickened im. 'Twas ther licker thet finerly fetched im all rite. Does yer feel all rite now—honey?'' she asked her spouse. On his favorable reply, she told us to ''give both of us er nother swaller of ther liker and you kin finish with me.'' We did so and she resumed the chair and she went through to the finish of the extracting, without a murmur. In course of time we rehabititated her mouth with store teeth. Months afterwards she met us and said they was fine and don't you know they air plaging ther life outen hus—bun fer faintin jest ter git er chance at that are bottle er licker you had.

---

He was a veteran of the war, from the lowly order of society—a son of the soil. We had been his dentist for years and he was faithful to us in the extreme.

He had lost his wife and called on us to have a tooth extracted and to tell us of his loss and distress. "Hits alrite now Doc, hits been er botherin' me fur sum time an' I jest thought I'd let yer jerk hit. Yes, I lost mer wife, an er mity good un she were. Hits true we useter fuss at times, but she wus nachuly er good woman an I misses her. But I kaint grieve allus, I hev ter have er helpmate, an er companion es ther good bock seys, an I've got mer eye on a mity likely widder in my naborhood, Mis Brown. She's got er leetle farm an sum spare cash an only one chile, er son, nigh bout grown—I think I'll take her in."

We met him a few months after this in a new buggy, all spruced up and beside him sat a commonplace woman of middle age, dressed in all the hues of color. "Yes, Doc, this is mer new wife. Hits our fust trip ter town and we wus agoin ter call on yer, fer she wants er set er teeth, but hits so late now we'll put hit off till later.

Time passed on until several months had rolled around, we had almost forgotten him and his affairs in our recurring days of practice, when he appeared at our office to have another sore tooth jerked! "No use ter ask me how mer wife is for I'm wuss off now than wen I had tuther tooth tuk out. I'm er grass widerwer now. Wese separated een ritin accordin ter agreement but the law in our state, yer know, don't alow eny divorce, an I kain't try ernother one. Oh she wus er fusser from er way back and I wus small pertaters erlong side that boy er hern. I was er goin' to present her with them teeth we wus ertalkin about, but she begun shortly atter that, to kick up so onnuchul I jest shut down on her, an we jest had it ontil we parted, es I told you. Well I do miss my ole Mandy, that us mer fust wife. I uster, during her life time, ter say ter fokes, that my opinion uv wimmin wus "God bless em" an' "Plague take em." Well, now, the fuss is fer Mandy and the las is fer Miss Brown, as she calls herself. I mus be ergoin ter look after mer lonely house. Good by." The widow had taken him in.

